# Effect on thermal stability of microstructure and morphology of thermally-modified electrospun fibers of polybenzoxazines (PBz) blended with sulfur copolymers (SDIB)

**DOI:** 10.1039/d1ra00705j

**Published:** 2021-03-09

**Authors:** Ronaldo P. Parreño, Ying-Ling Liu, Arnel B. Beltran

**Affiliations:** Department of Chemical Engineering, De La Salle University 2401 Taft Avenue Manila 1004 Philippines arnel.beltran@dlsu.edu.ph; Chemicals and Energy Division, Industrial Technology Development Institute (ITDI), Department of Science and Technology (DOST) Taguig 1631 Philippines ronaldo_parrenojr@dlsu.edu.ph rpparrenojr@yahoo.com; Department of Chemical Engineering, National Tsing Hua University Hsinchu 30013 Taiwan liuyl@mx.nthu.edu.tw; Center for Engineering and Sustainable Development Research, De La Salle University 2401 Taft Avenue Manila 1004 Philippines

## Abstract

Simple modification by thermal treatment is the commonly used approach to enhance the performance of electrospun fibers. This was investigated in the thermal treatment of polybenzoxazine (PBz) fibers blended with sulfur copolymers (SDIB) to determine the effect of varying treatment conditions on the microstructure and morphology of PBz fibers with the effect of incorporating sulfur functional groups on resulting properties. Mechanical properties of PBz are greatly improved by thermally-induced ring-opening polymerization (ROP) of the oxazine ring. Blending with sulfur copolymers (SDIB) could have beneficial effects on endowed features on fibers but could also affect the resulting properties of SDIB-blended PBz fibers during crosslinking reactions. Fiber mats were fabricated by electrospinning of PBz (10 wt%) blended with SDIB (10 wt%). Physical modification with varying conditions of sequential thermal treatment were evaluated and compared to the conditions applied on pristine PBz fibers. Changes in morphology and microstructure of fibers after modification were analyzed through scanning electron microscopy (SEM) while elemental compositions were identified after varying the conditions of thermal treatment. Adjustment of treatment conditions using two-step temperature sequential thermal treatment with higher temperatures of 160 °C and 240 °C showed significant changes in microstructure and morphology of fibers. Lower temperatures of 120 °C and 160 °C exhibited microstructure and morphology of fibers which affected the fiber diameter and fiber networks. Cross-sectional SEM images also confirmed the adversed effect of high-temperature treatment conditions on fibrous structures while low-temperature treatment retained the fibrous structures with more compact and stiff fiber networks. SDIB-blended PBz fibers were also evaluated by TGA and DSC to correlate the changes in structure and morphology with the thermal stability and integrity of blended SDIB/PBz fibers as compared to pristine PBz with the effect of change in treatment conditions. Fiber strength indicated slower weight loss for blended fibers and higher onset temperature of degradation which resulted in more thermally stable fibers.

## Abstract 2

Microstucture and morphology of thermally-modified electrospun fiber of PBz blended with SDIB that enhanced structural satbility and integrity.

## Graphical abstract

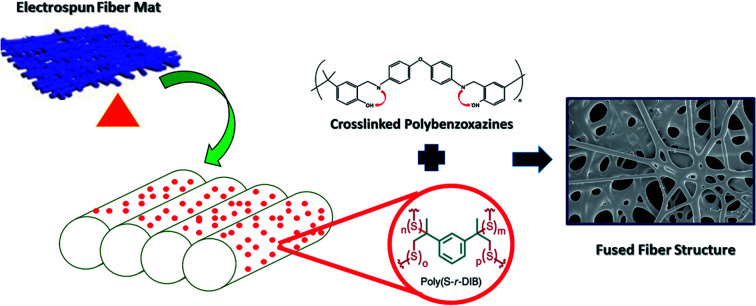

## 1. Introduction

Nanofibers (NF) have been gaining lots of interest in nanoscience and nanotechnology, being an important one-dimensional (1D) nanostructured-material that can provide custom-made properties for high performance materials.^1^ This is in addition to carbon nanotubes (CNTs) which are a well-known class of 1D carbon nanomaterials^2^ that are currently being explored for the development of new electrocatalyst materials for processes such as hydrogen and oxygen evolution for renewable power generation.^3,4^ The unique and excellent features make electrospun nanofibers a class of nanomaterials highly suited for a wide range of applications, such as “smart” mats, filtration membranes, catalytic supports, energy harvesting/conversion/storage components, and photonic and electronic devices, as well as biomedical scaffolds.^5^ Other new but very significant emerging applications at this time with global concerns for the environment are nanofibrous membranes for air filtration with superior photocatalytic and antibacterial activities,^6,7^ water purification^8^ and oil/water separation with antibacterial and self-cleaning properties.^9^ The unique functional properties are derived partly from the fabrication method using electrospinning (ES) that formed fibers possessing high specific surface areas and porosities, controllable compositions,^10^ higher aspect ratio and better pore interconnectivity.^11^ These significant features made ES a versatile and viable technology for the production of nanofiber-based materials.^5^ The versatility of ES allows the formation of nanofibers with different morphologies and structures by controlling process parameters such as applied voltage, tip-to-collector distance, collector type, nozzle design and feed rate.^11^ It is also recognized as an easy method compared to other standard conventional fiber preparation methods that can utilize natural and synthetic polymers, polymer blends, composites with metal or ceramic particles and nanocomposites.^12^ These unique functionalities achieved from electrospinning new and promising materials quickly found its way for new and emerging applications. But with the demand for high-performance materials is the need to modify properties of nanofibers to suit required specific end-use properties. Physical modification is one of the most efficient strategies utilized to enhance the fiber structure and morphology of electrospun nanofibers after electrospinning. Modification of electrospun fibers after electrospinning is called post-electrospinning treatment which is more preferred for its efficiency to provide the required modification.^13^ Depending on the polymer type and required properties of the material, the suitable treatment should provide modification and enhancement after curing of the polymeric fibers.

**Fig. 1:**
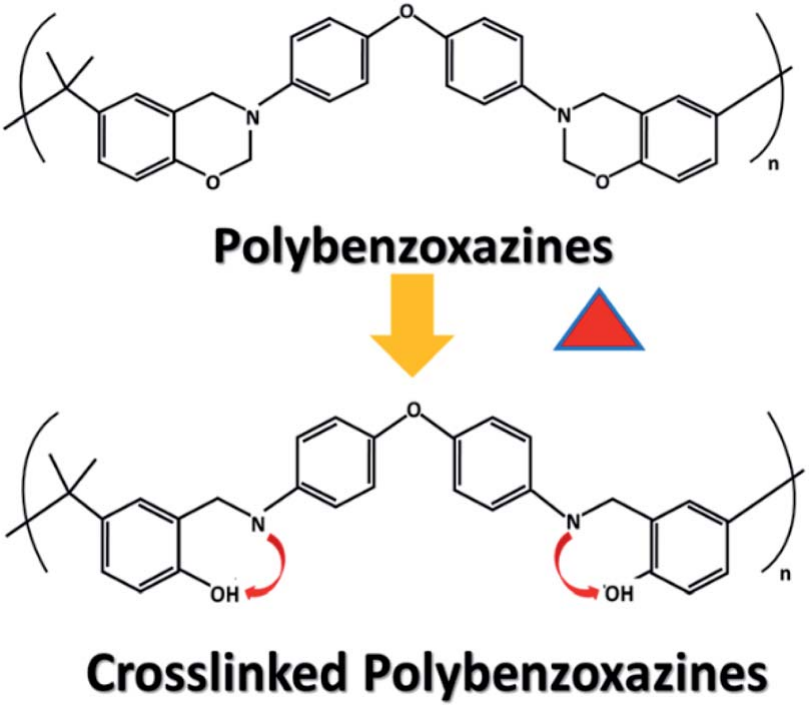
Crosslinking *via* activated ring-opening polymerization of the oxazine ring of PBz.

New polymers such as polybenzoxazines (PBz) is currently being studied by preparing modified monomers with additional functionality or blended with another polymer.^14^ PBz is a superior alternative to thermoset-type polymers for high performance applications.^15^ In the work of Li and Liu,^16^ the post-electrospinning treatment of fiber mat of PBz was carried out *via* thermal treatment. PBz when thermally treated at elevated temperature results to crosslinking *via* thermally activated ring-opening polymerization (ROP) of the oxazine ring in the main polymer chain (Fig. 1).^16,17^ This post-treatment produced high-performance PBz polymer with combined thermal and mechanical properties.^18^ Thermal curing of PBz occurs without hardeners or catalysts,^19^ but the high temperature requirement of at least 200 °C for crosslinking is its major limitation to attain stability. Much interests of previous researchers dealt with the molecular structure of PBz with its design flexibility so different synthetic methods for the preparations of benzoxazine monomers and blends was carried out.^20^

In this study, polybenzoxazines (PBz) blended with sulfur copolymers (SDIB) were fabricated into electrospun fibers. Additional functionality from sulfur groups was examined by blending of SDIB with PBz to possibly endow new features on fiber structure and morphology. This investigated the effects of sulfur groups on crosslinking reaction of blended PBz and how electrospun fibers responded with the varying treatment conditions. Polymer blending is known as an easy and cost-effective method of developing new polymeric materials from mixing two or more polymers with synergistic properties manipulated by the component polymer.^21^ After processing into fibers, post-electrospinning thermal treatment was analyzed for its effects on curing of fibers. Hence, the blending of PBz with SDIB synthesized from inverse vulcanization of elemental sulfur (S_8_) provided new insights on the influence of changing treatment conditions on fiber morphology, structures and thermal stability which can be further optimized to provide solution on the enhanced curing condition of PBz with other polymers in polymer blend. Simple modification of electrospun PBz fibers blended with polymer as the main focus of this research could provide new perspective on treatment conditions for improved structural composition and stability of fibers of PBz for its intended applications.

## 2. Experimental section

### 2.1 Materials

Sulfur (S_8_) (powder, sublimed, Alfa Aesar 99.5%) and 1,3-diisopropenylbenzene (DIB) (TCI Chemicals, >97.0%) were used as received without any purification. Sulfur copolymers (SDIB) were synthesized in the lab based on the procedure described in the works of Chung *et al.*^22^ S_8_ and DIB mass ratio used in the synthesis was 50/50 wt/wt% as previously studied in the works of Parreño *et al.*^23^ Polybenzoxazines (PBz) was prepared in the lab as described in the works of Lin *et al.*^24^ Dimethyl sulfoxide (DMSO) (ACS grade, Echo, 99.9%), and tetrahydrofuran (THF) (inhibitor free high purity, Tedia, 99.8%) were used as received.

### 2.2 Electrospinning of SDIB-blended PBz fiber mat

PBz (10 wt%) blended with SDIB (10 wt%) was prepared by using mixture of DMSO and THF (1/3) (v/v) based on the procedure described in the works of Parreño *et al.*^25^ Electrospun fibers was fabricated using vertically-aligned electrospinning apparatus composed of a 10 ml syringe with needle (ID = 0.8 mm) connected to a syringe pump, a ground electrode, and a high voltage supply (Falco Enterprise Co. Ltd, Taipei, Taiwan). The needle was connected to the high voltage supply, which generates positive DC voltages up to 40 kV. Polymer solution of SDIB/PBz was placed in a 10 ml syringe which was ejected through a needle spinneret by a syringe pump with a mass flow rate of 1.00 ml h^−1^ and tip-to-collector distance (TCD) of 15 cm. Applied voltage was varied from 10 kV to 20 kV in the electrospinning process depending on the change in concentration of polymer blend while other process parameters were not changed. Electrospinning process was carried out at ambient conditions. After electrospinning process, the electrospun pure PBz (ES-PBz) and blended PBz/SDIB (ES-PBz/SDIB) fiber mats were set aside at ambient conditions for 24 h to vaporize remaining solvent prior to post-treatment.

### 2.3 Post-electrospinning thermal treatment of PBz/SDIB fibers

For pristine PBz (0% SDIB), the sequential thermal treatment was conducted at temperatures of 80 °C, 100 °C, 160 °C, 200 °C and 240 °C, each for 1 h inside the oven (Deng Yng, DH 400) to thermally crosslinked the PBz. Then, thermally cured mat was cool down to room temperature inside the oven and then, removed from the collector plate. For the electrospun fibers of PBz with 10% SDIB, the thermal treatment was initially tested based on the curing conditions used for pristine PBz but showed unsatisfying results. Consequently, the suitable treatment conditions were examined to determine the influence on the final properties of electrospun fibers. The adjusted thermal treatment condition was changed to only two-step temperature after examining the microstructure and morphology of cured fibers. The relative fiber mass yield measured from the non-woven fibers produced by electrospinning of polymer blend solution after thermal treatment were approximately 0.98 g for ES-PBz (10 cm × 10 cm; average thickness: 103 μm ± 15) and 0.94 g for ES-PBz/SDIB (10 cm × 12 cm; average thickness: 74 μm ± 18).

### 2.4 Characterization of electrospun fibers

Evaluation of electrospun fibers were conducted to characterize the resulting properties of PBz blended with SDIB fibers and to understand the effect of the thermal modification on structure and morphology. SEM-EDX analysis was carried out with a Scanning Electron Microscope (SEM) (FEI Helioz Nanolab 600i, Eindhoven, The Netherlands) with Energy Dispersive X-ray Spectroscopy (EDX) (Oxford Instrument X-Max, Abingdon, U.K.) at the Advanced Device and Materials Testing Laboratory (ADMATEL) of DOST. Freeze fracture using nitrogen has been used to prepare cross-sections of electrospun fiber mats for SEM imaging analysis. Thermal behavior and property of the fiber mats were measured by differential scanning calorimetry (DSC) using a Q20, TA instrument at heating rate of 10 °C min^−1^ in the temperature range of 50–300 °C under nitrogen environment. Thermal stability was determined using thermogravimetric (TGA) analysis which was carried out in platinum pans using a Q50 IR analyzer (TA instruments) with an automated vertical overhead thermobalance. The samples were heated at 10 °C min^−1^ to 800 °C under nitrogen gas.

## 3. Results and discussion

### 3.1 Fabrication of PBz blended with SDIB fibers

**Fig. 2:**
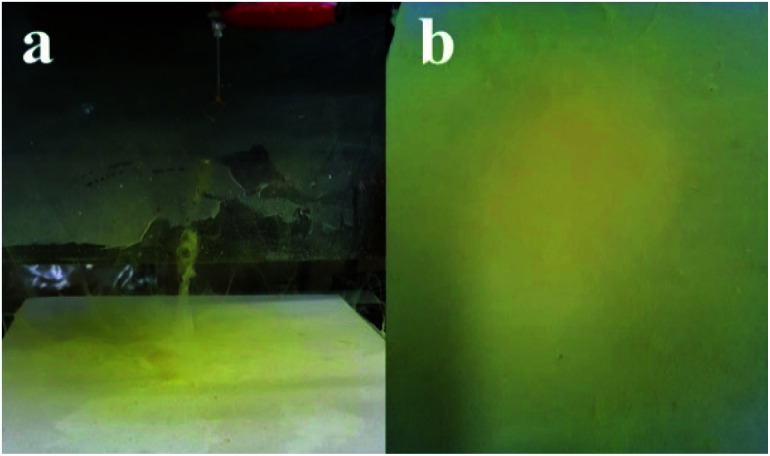
PBz/SDIB showing fiber formation (a) ejected from the tip of the needle and (b) collected in the grounded plate.

PBz blended with SDIB was processed *via* electrospinning process. Solution concentration is a polymer property which was inherent on the copolymers–polymer interaction. This was considered in electrospinning process by allowing manipulation of applied voltage in order to counteract the change in polymer properties with the addition of SDIB to PBz. Other process parameters such as tip-to-collector distance, needle size and flow rate were not changed during the process. Pure PBz electrospun fibers were obtained by using an applied voltage of 10 kV. This became the basis for the initial runs of SDIB-blended PBz using the same applied voltage but further increased to an applied voltage of 20 kV in the subsequent run resulting to formation of fibers in the grounded plate collector. The ejection of the polymer jets from the apex of the Taylor cone formed at the tip of the needle is achieved with further increase in voltage beyond a critical value.^26^ At voltage level of 20 kV, the critical value of voltage needed for PBz blend was attained to overcome the effect of polymer concentration and produced polymer jets at the tip of needle to form fibers in the collector plate as shown in Fig. 2a. The mat was obtained after continuous electrospinning for at least 4 h as shown in Fig. 2b. The yellowish color of the fibers forming at the tip of the needle as well as the fiber mat collected in the plate indicated that SDIB was incorporated in the fibrous material.

### 3.2 Modification by thermal treatment

**Fig. 3:**
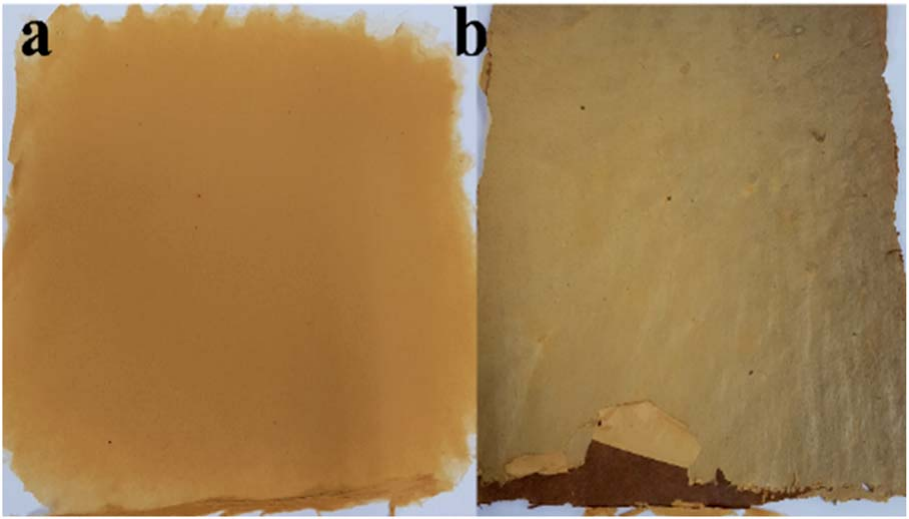
Thermally-modified electrospun fibers (a) pure PBz and (b) PBz blended with SDIB.

The modification by thermal treatment on fibers after electrospinning process was evident on the fiber structure, morphology and physical appearance. For ES-PBz, the post-thermal curing was conducted to effectively crosslinked the fiber networks according to the conditions in the works of Li and Liu.^16^ However, the thermal treatment conditions for ES-PBz/SDIB were not yet established so initial test run was based on the thermal curing conditions of ES-PBz. The fiber mats of ES-PBz and ES-PBz/SDIB as shown in Fig. 3 exhibited the results of sequential thermal curing of up to temperature of 240 °C. The ES-PBz fiber mat was effectively thermally cured with smooth and even dark brown surface at this condition in Fig. 3a. However, for ES-PBz/SDIB, the fiber mat showed the effect of very high treatment temperature of 240 °C which was evident in the color and texture of the fibers with uneven darker surface areas in Fig. 3b. It was also observed that the mat easily disintegrates when removed from the collector.

**Fig. 4:**
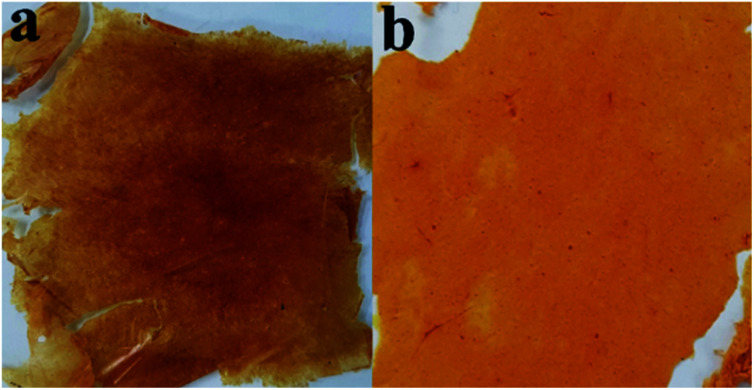
ES-PBz/SDIB thermally treated at two-step sequential temperature of (a) 160 and 240 °C and (b) 120 and 160 °C.

In the subsequent runs, the thermal modification condition was changed to determine the effect of varying temperature on the fiber mat's appearance and properties. Two-step temperature sequential thermal treatment was then applied to the fiber mat to evaluate the effect of shorter time of treatment. The first condition was carried using temperatures of 160 °C and 240 °C each for 1 h based on the temperature requirement for thermal crosslinking of PBz. The change of treatment condition to just two-step sequential temperature but retaining the same final temperature of 240 °C exhibited similar effect on the fiber mat from previous treatment as shown in Fig. 4a with uneven darker brown-coloured surface areas and tattered mat showing sign of over cured and more brittle mat. Based on this result, thermal treatment condition was changed by lowering the temperatures to 120 °C and up to 160 °C only each for 1 h. A better fiber mat was obtained from lowering the treatment temperature as shown in Fig. 4b. The mat showed more uniformed light brown-coloured surface area which was compact and durable as compared to the other mat which easily disintegrates. This was an indication of better post-electrospinning treatment condition for ES-PBz/SDIB as compared to the first modification condition.

### 3.3 SEM-EDX analysis of thermally-modified fibers

**Fig. 5:**
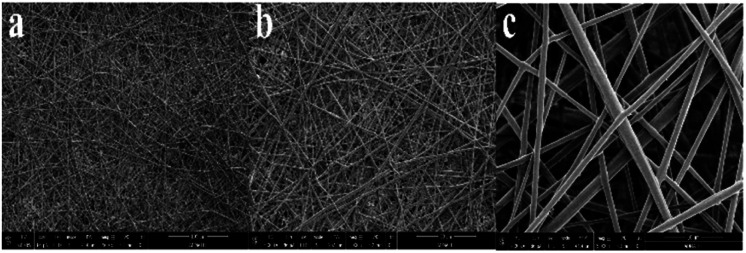
SEM images of ES-PBz fiber mat at scale bar of (a) 100 μm, (b) 50 μm and (c) 10 μm; (magnification: 500×, 1000× and 5000×).

The influence of thermal treatment conditions with varying temperatures on the evolution of the structure and morphology was examined by analyzing the SEM images. These changes from undergoing different sequential temperatures of thermal treatment clearly indicate the effect of incorporating SDIB in the polymer blend when formed into fibers. For ES-PBz fiber mat, the SEM images in Fig. 5a–c showed the expected structure and morphology of randomly-oriented continuous, interconnected fibrous structures with highly uniform and smooth fibers after thermal treatment of as-spun fibers.

**Fig. 6:**
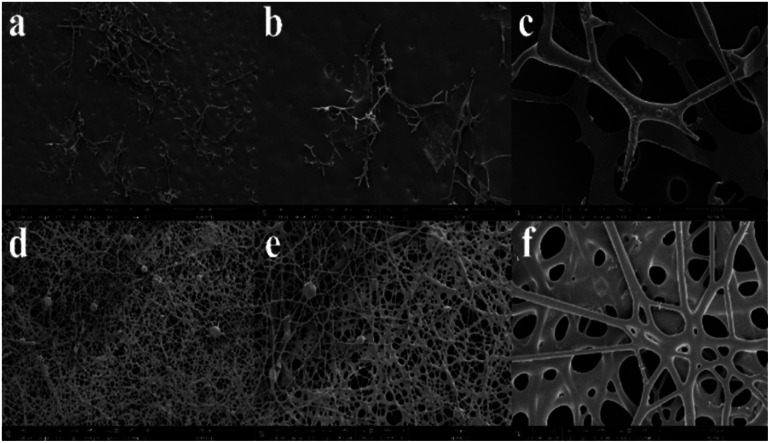
Thermally-treated ES-PBz/SDIB SEM images at temperatures of (a) 160 °C and 240 °C (scale bar:100 μm), (b) 160 °C and 240 °C (scale bar: 50 μm), (c) 160 °C and 240 °C (scale bar: 10 μm), (d) 120 °C and 160 °C (scale bar: 100 μm), (e) 120 °C and 160 °C (scale bar: 50 μm), and (f) 120 °C and 160 °C (scale bar: 10 μm); (magnification: 500×, 1000× and 5000×).

Changing the treatment condition with two-step temperature sequential treatment but retaining the temperatures of 160 °C and 240 °C caused drastic impact on the fiber structure and morphology based on the SEM images as shown in Fig. 6a–c. It showed that fibrous structures and morphology of ES-PBz/SDIB fibers were lost due to aggregation and melting of fibers. This occurrence was an indication of over curing where the functional group of SDIB contributed to the lowering of temperature requirement for thermal curing of PBz when blended. Lowering the temperature in the second condition during thermal treatment showed the influence of SDIB in retaining the morphological and structures of the fiber networks which was almost similar to ES-PBz as shown in Fig. 6d–f. It was observed from the results that the fiber mat exhibited almost the same randomly-oriented continuous, interconnected fibers with occurrence of conglutination in fibrous structures. The presence of conglutination is the results of partial solidification of jets which can produce fibers attached at points of contact which stiffen the mat due to strong attachments at crossing points.^20,27^ In this case, due to heat treatment of fiber mat, the SDIB added to PBz soften (the *T*_g_ of pure SDIB being an amorphous glass is 22–24 °C) and created conglutinate fibers at certain locations which strengthen the thermally-treated fiber mat. Although the mat has uniform and smooth fibers but few beads-on-string fibers occurred due to the application of higher applied voltage. Based on these results, the addition of SDIB lowered the required temperature for thermal treatment of polymer blend of PBz which have almost similar morphology and structures and even enhanced the strength of the mat by heat treatment to 120 °C and 160 °C due to the conglutination on fiber networks.

SEM images were evaluated for fiber diameter and pore size of fibers and compare the influence of the thermal modification on ES-PBz and ES-PBz/SDIB. For ES-PBz fibers having a more uniform fiber morphology, the average fiber diameter is 2.67 ± 0.99 μm. The results of blending SDIB on PBz fibers exhibited a slight increase in fiber diameter by 0.50 μm with average fiber diameter of 3.07 ± 1.35 μm. This is mainly due to the concentration effect of adding SDIB which required the application of higher applied voltage during electrospinning. The effect on fiber morphology of post-electrospinning thermal treatment was clearly shown in the average pore size of the electrospun fibers. SEM images revealed almost the same structures of randomly-oriented continuous, interconnected fibers for both ES-PBz and ES-PBz/SDIB but with the presence of conglutinate fibers on ES-PBz/SDIB which resulted to difference in pore size. For ES-PBz fibers, the average pore size is 0.29 μm while the average pore size of PBz/SDIB fibers increased to 0.45 μm. The increased in mean fiber diameter and occurrence of conglutinate fibers after thermal treatment contributed to the change in pore size of PBz blended with SDIB fibers. The results are summarized in Table 1.

**Table 1:** Fiber diameter and pore size electrospun fibers

Electrospun fiber	Average fiber diameter, μm	Average pore size, μm
ES-PBz	2.67 ± 0.99	0.29
ES-PBz/SDIB	3.07 ± 1.35	0.45

**Fig. 7:**
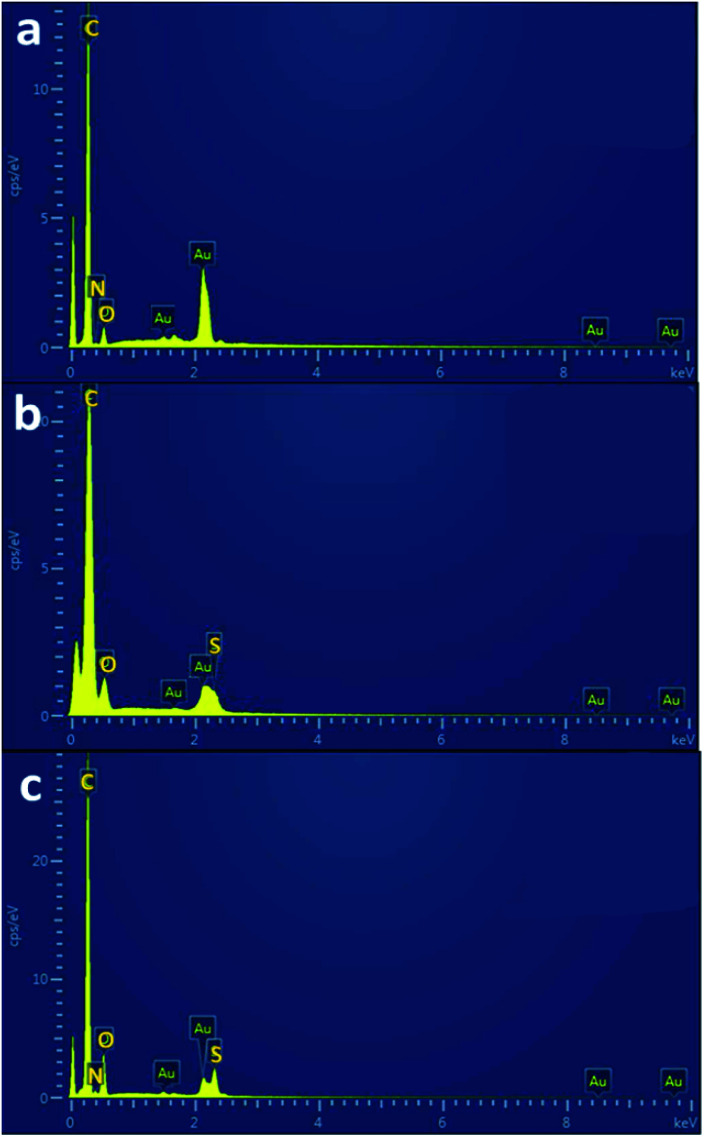
EDX spectra of thermally-modified fiber mats of (a) ES-PBz, (b) ES-PBz/SDIB at temperature treatment conditions of 160 °C and 240 °C, and (c) ES-PBz/SDIB at temperature treatment conditions of 120 °C and 160 °C; other peaks in the spectra are Au used as coating material.

Energy Dispersive X-ray (EDX) analysis was used to identify the elemental compositions of the electrospun fiber materials after undergoing varied conditions of thermal treatment. These results were also confirmation of the presence of sulfur groups from SDIB in the PBz fibers. The fiber mats of PBz blended with SDIB at two different sequential thermal treatment conditions were analyzed for the presence of elements which were compared to pure ES-PBz fiber mat. Based on the EDX spectra in Fig. 7a, the elemental components of the pure ES-PBz fibers were predominantly carbon element with oxygen and nitrogen. Comparing these results as bases for the effect of blending on ES-PBz/SDIB at two-step temperature treatment condition of 160 °C and 240 °C, Fig. 7b showed carbon and oxygen elements with the sulfur groups that confirms the successful incorporation of SDIB in the fibers. However, the absence of nitrogen element was also observed which could be attributed to the effect of high temperature treatment condition of up to 240 °C which caused the melting of fibers. The reason for this occurrence could be linked to the degradation of the amine parts in the crosslinked structure of PBz. According to Ran *et al.*,^28^ the amine part is easier to cleave during degradation due to the weak C–N bond which can also be observed as weight loss of the aromatic amine in the thermogravimetric analysis. For the lower two-step temperature treatment condition of 120 °C and 160 °C in Fig. 7c, it revealed the same elemental compositions of carbon, oxygen, sulfur and the retention of nitrogen which proved that lowering the temperature of thermal treatment benefited the microstructure and morphology as well as the stability of the fibers.

The elemental compositions with the corresponding percentages were summarized in Table 2. These results further verified the applicability of the thermal treatment condition where the nitrogen retained in the lower temperature condition was almost the same with the nitrogen composition of pure ES-PBz. For the sulfur groups, lesser amount was observed in the higher temperature conditions as compared to the lower treatment condition which was due to the effect of thermal degradation where sulfur elements were also lost along with the degradation of amine parts.

**Table 2:** Elemental analyses of fiber mats at different treatment conditions

Electrospun fiber	% C	% O	% N	% S
ES-PBz	84.4	9.5	5.5	—
ES-PBz/SDIB (160 and 240 °C)	85.1	11.1	—	3.4
ES-PBz/SDIB (120 and 160 °C)	75.8	13.1	5.1	5.7

**Fig. 8:**
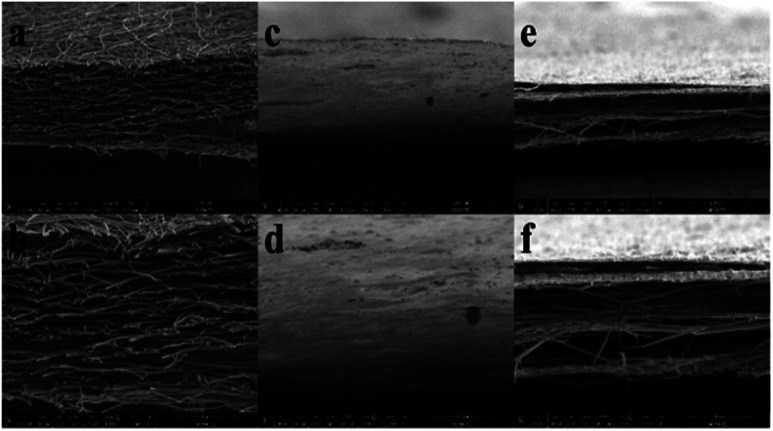
Cross sectional SEM images of ES-PBz at scale bar of (a) 100 μm, (b) 50 μm; ES-PBz/SDIB first thermal treatment at scale bar of (c) 100 μm, (d) 50 μm; ES-PBz/SDIB second thermal treatment at scale bar of (e) 100 μm, (f) 50 μm; (magnification: 500× and 1000×).

The cross-sectional SEM images of the electrospun fiber mats were also analyzed for the two thermal treatment conditions and compared with the ES-PBz fiber mat. In Fig. 8a and b, the cross-sectional images of pure PBz fibers revealed the presence of stack of fiber networks with randomly-oriented interconnected fibers. For the first thermal treatment condition, the cross-sectional images in Fig. 8c and d showed no fiber structure which was caused by the melting and aggregation of fibers due to over curing of the mat. In the second thermal treatment condition, Fig. 8e and f showed layers of fibers which retained similar cross-sectional structure of pure PBz fiber mat.

Although the mat was more compact and fiber network are more attached with each other, this was the confirmation of the conglutination that occurred. These results verified that the microstructures of the electrospun fibers with the addition of SDIB showed almost similar morphology and fiber structure when thermally treated at lower temperature than the thermal crosslinking condition for pure electrospun PBz fibers.

### 3.4 Thermal property and stability of PBz/SDIB fibers

**Fig. 9:**
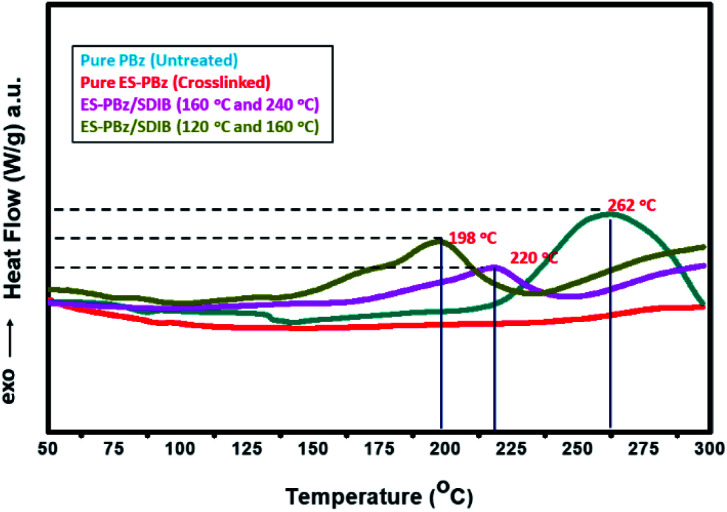
DSC profile of pure PBz, thermally-modified ES-PBz and ES-PBz/SDIB fibers.

Thermal treatment conditions were evaluated for its effect on the degree of curing of the fibrous mat of PBz/SDIB using DSC analysis. The DSC profiles of pure PBz and thermally-modified pure ES-PBz and ES-PBz/SDIB are presented in Fig. 9 to observe the thermal behavior and compares the thermal properties of pure materials with the fibers of polymer blend. For untreated pure PBz, it showed a large exothermic peak at temperature of 262 °C as the maximum rate of curing. This means that the polymer had not undergone crosslinking and was not completely cured. Comparing this to thermally-treated pure ES-PBz, there was no heat of cure observed which indicated that the fiber mat was completely cured and became crosslinked. In the case of the thermally-treated ES-PBz/SDIB, both DSC profiles showed exothermic peak of curing but with different degrees in relation to the size of peak. It is observed that for thermoset resins like PBz, as it becomes more crosslinked, the residual heat of curing as a measure of degree of curing, decreases in size and becomes undetectable when the material is completely cured.^29^ For ES-PBz/SDIB which was thermally-modified at higher temperature of 160 °C and 240 °C, the DSC profile revealed that the heat of curing was relatively smaller compared to both ES-PBz/SDIB treated at lower temperature and untreated pure PBz. This showed that in terms of degree of curing, it was more cured and crosslinked. However, the onset temperature of cure which occurs when the heat flow deviates from the linear response was higher with exothermic peak temperature of 220 °C. For the thermally-modified ES-PBz/SDIB at lower temperature of 120 °C and 160 °C, the exothermic peak of curing was relatively larger compared to the ES-PBz/SDIB treated at higher temperature but still smaller than the pure untreated PBz. The significant impact of this lower curing temperature was the lower onset temperature of curing with significantly lower exothermic peak temperature of 198 °C. Although the fiber mat was not 100% cured, at this curing condition the thermal degradation between 200–300 °C due to loss of aromatic amine was avoided.^28^ This thermal treatment condition prevented the melting of fibers and complete loss of microstructures but more importantly, contributed in enhancing the stiffness and strength of the fiber networks. Another important benefit of this lower thermal treatment condition is its effect on the structural stability and mechanical integrity of the fiber mat. The degree of curing for materials containing thermoset resins is directly related to brittleness, impact resistance, long term stability, creep, solvent resistance and product integrity.^30^ It was found out that more highly-crosslinked material resulted to a more brittle material because of the high crosslinking which lowers the segmental mobility.^20^

**Fig. 10:**
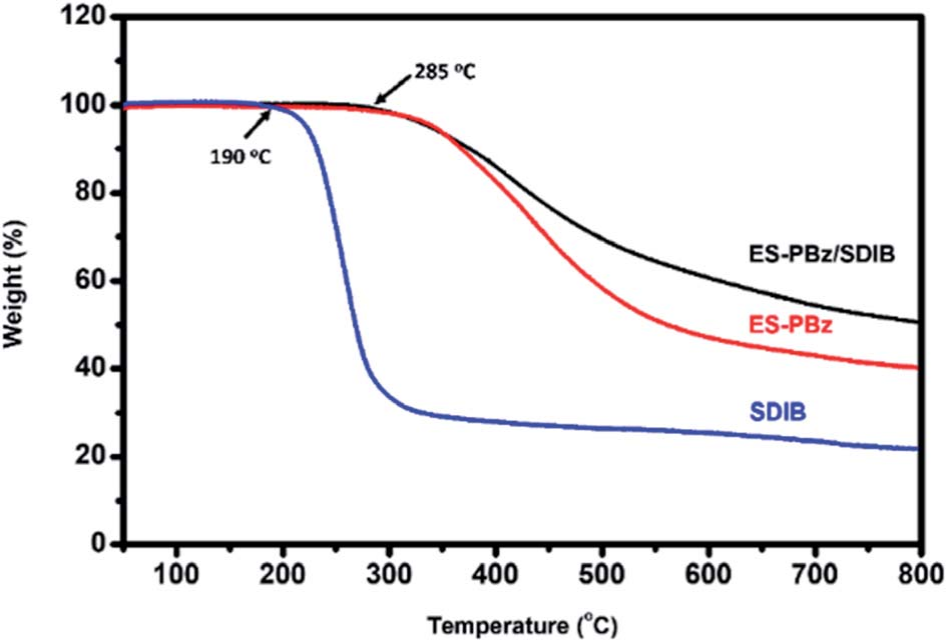
Thermal stability of pure SDIB, ES-PBz fiber mat and ES-PBz/SDIB fiber mat.

Thermal stability of fiber mat is a critical factor in its final application as membrane for various separation and adsorption processes as well as for emerging applications, so modification has to be done for tuning of its properties including the degradation rate of the material. The modification of electrospun fiber mat is carried out by post-electrospinning treatment to effectively modify its properties. For ES-PBz and ES-PBz/SDIB, the thermal treatment resulted to enhanced thermal stability. Based on the TGA thermograms as shown in Fig. 10, pure SDIB, ES-PBz and ES-PBz/SDIB all showed single-step degradation. The onset temperature of degradation for pure SDIB is at 190 °C which is much lower than the ES-PBz and ES-PBz/SDIB fiber mats which both have higher onset temperature of degradation of 285 °C. But comparing the ES-PBz and ES-PBz/SDIB, slower weight loss was observed with the addition of SDIB and thermally-treated at lower temperatures. A significant amount of ES-PBz/SDIB fiber mat with 55% of weight still remaining after reaching the temperature of 800 °C while only 42% for ES-PBz fiber mat. The incorporation of SDIB to PBz and application of thermal treatment at lower temperatures both contributed to slower degradation rate and more thermally stable fibers of ES-PBz/SDIB.

The structural stability enhancement in the electrospun fiber mat was attained by the modification in morphology and microstructure. Electrospun fibers are randomly distributed, non-interconnected fibrous structures prior to treatment which produces a weaker material. The poor mechanical strength of electrospun fibers are mostly due to the higher porosity and even worsened by weak bonding at fiber junctions but post-treatment helps in welding the fibers and strengthened the junction points without greatly affecting the morphology and microstructure.^31^ The application of thermal treatment of fiber mat helps to form fused fibrous structures which enhanced the structural integrity and mechanical strength.^13^ But in addition to application of heat, the SDIB also provided its effect on the structural changes of the fibers. It was found out that incorporation of other functionalities can influence the curing behavior of PBz which would result to a different morphology and microstructure and consequently, improving the mechanical and thermal properties of the cured material.^20^ This was revealed in the PBz fibers with added sulfur functionalities that resulted to fibers with conglutination in structure. These structural changes produced fibers attached at points of contact which contributed to fibers' stability and integrity.

## 4. Conclusions

Post-electrospinning thermal modification condition applied to electrospun fibers from the polymer blending of SDIB with PBz has demonstrated the significant contribution of high-temperature sequential treatment for fiber structures and morphology. Adjusting a more suitable thermal treatment condition at low-temperature sequential treatment in consideration of polymer blend compositions retained the same morphology and structure of fibrous PBz. Observable significant changes occurred with the presence of conglutinate fibers that contributed in strengthening the fiber networks for a more stable mat. This result was confirmed by the TGA showing higher onset temperature of degradation with slower weight loss and more thermally stable PBz/SDIB fibers than pure PBz. The DSC profile also confirmed the contribution of curing at lower temperature to enhance strength and stability of the fibers. The fiber diameter and pore size also exhibited the influence of blending SDIB in lowering the temperature of thermal treatment which could be further explored for other properties. Thus, the incorporation of sulfur functional groups in electrospun fibers of PBz using SDIB influences the curing behavior of benzoxazines and result to changes in microstructure and morphology which consequently affects other properties such as thermal stability. The optimization of electrospinning process of PBz blended with SDIB and the temperature of sequential treatment could have the potential of producing a high-performance material for membrane separation, adsorption and purification processes as well as other emerging applications in future research works.

## Author contributions

Conceptualization, R. P. P. J. and A. B. B.; methodology, R. P. P. J., Y.-L. L. and A. B. B.; formal analysis, R. P. P. J., Y.-L. L. and A. B. B.; investigation, R. P. P. J., Y.-L. L. and A. B. B.; data curation, R. P. P. J.; resources, Y.-L. L.; writing—original draft preparation, R. P. P. J.; writing—review and editing, R. P. P. J. and A. B. B.; supervision, Y.-L. L. and A. B. B.; funding acquisition, R. P. P. J. and A. B. B.

## Conflicts of interest

The authors declare no conflict of interest.

## Supplementary Material
